# Global Diversity of the Brachypodium Species Complex as a Resource for Genome-Wide Association Studies Demonstrated for Agronomic Traits in Response to Climate

**DOI:** 10.1534/genetics.118.301589

**Published:** 2018-11-16

**Authors:** Pip B. Wilson, Jared C. Streich, Kevin D. Murray, Steve R. Eichten, Riyan Cheng, Nicola C. Aitken, Kurt Spokas, Norman Warthmann, Sean P. Gordon, Accession Contributors, John P. Vogel, Justin O. Borevitz

**Affiliations:** * The ARC Centre of Excellence in Plant Energy Biology, Research School of Biology, Australian National University, Canberra, Australian Capital Territory 200, Australia; † Department of Psychiatry, University of California San Diego, La Jolla, California 92093; ‡ Ecogenomics and Bioinformatics Lab, Research School of Biology, Australian National University, Canberra, Australian Capital Territory 200, Australia; § Soil and Water Management, Agricultural Research Service, United States Department of Agricutlture (USDA), St. Paul, Minnesota 55108; ** Department of Energy, Joint Genome Institute, Walnut Creek, California 94598

**Keywords:** population genetics, climate change, agronomic traits, climate simulation, genome-wide association studies, ecogenomics, Brachypodium distachyon, genotyping, plant physiology

## Abstract

The development of model systems requires a detailed assessment of standing genetic variation across natural populations. The Brachypodium species complex has been promoted as a plant model for grass genomics with translation to small grain and biomass crops. To capture the genetic diversity within this species complex, thousands of Brachypodium accessions from around the globe were collected and genotyped by sequencing. Overall, 1897 samples were classified into two diploid or allopolyploid species, and then further grouped into distinct inbred genotypes. A core set of diverse *B. distachyon* diploid lines was selected for whole genome sequencing and high resolution phenotyping. Genome-wide association studies across simulated seasonal environments was used to identify candidate genes and pathways tied to key life history and agronomic traits under current and future climatic conditions. A total of 8, 22, and 47 QTL were identified for flowering time, early vigor, and energy traits, respectively. The results highlight the genomic structure of the Brachypodium species complex, and the diploid lines provided a resource that allows complex trait dissection within this grass model species.

CLIMATE change is impacting the production of food worldwide (Wheeler and von Braun 2013), and increasing global demand will soon outstrip the rate of improvement in crop yield by traditional breeding methods (Ray *et al.* 2013). To address food and climate security, there is a need for agricultural innovation across a range of scientific disciplines, from genomics to phenomics in new species across the landscape (Rivers *et al.* 2015). Breeding for more variable future climates, and for broad adaptability, requires an understanding of the plasticity of the genetic architecture of agronomic traits across environments. The use of dynamic climate chambers, that can mimic regional diurnal and seasonal climate types (Brown *et al.* 2014), allows us to examine the genetic architecture underlying complex adaptive traits across field-like environments.

Three complex traits that have a large impact on yield are ear emergence, early vigor, and energy use efficiency. The timing of ear emergence is crucially important to yield in many grain-growing regions, including Australia, where early flowering may lead to cold-induced sterility, while late flowering may result in heat stress or lack of water-limiting grain filling. Early vigor, defined as an increase in the above-ground biomass prior to stem elongation, is a beneficial trait in many environment types, especially when combined with increased transpiration efficiency (Condon *et al.* 2004). Since vapor pressure is low in winter, increased biomass during early growth improves plant water use efficiency. Early vigor also increases competition against weeds, reduces soil evaporation and may improve yields by increasing total seasonal biomass (Wilson *et al.* 2015a). Energy use efficiency is a relatively understudied component of plant growth that represents the efficient transfer of energy, acquired through photosynthesis, to the grain, and may significantly affect yield. Early studies indicate that energy efficiency, via lower respiration rates, is correlated with an increase in biomass in monocot species (Wilson and Jones 1982; Winzeler *et al.* 1988). Identification of the genetic architecture of energy use efficiency, timing of heading, and early vigor traits, as well as the genetic sensitivity to future temperature profiles, could accelerate breeding in crop species via selection for improved predicted yields in the future.

Genome-wide association studies (GWAS) combine dense genetic markers, identified via next-generation sequencing and high-throughput phenotyping, to identify the causative alleles and to predict complex quantitative traits (Atwell *et al.* 2010). The improvement of crop yield involves many complex traits, and the expression of these traits can be highly dependent on the growth environment. GWAS is an excellent method for mapping and predicting yield-related traits and their interaction with the environment. GWAS has been undertaken in a number of crop species; for dozens of agronomic traits in diploid species such as rice, barley, and corn (for review, see Huang and Han 2014), and has even be used reasonably successfully in wheat despite the added complexity of a hexaploid genome (*e.g.*, Sukumaran *et al.* 2014).

*Brachypodium distachyon* is a model species for temperate C3 grass crops such as wheat, barley, rye, and oats as it is also located in the Pooideae family and has a number of advantageous characteristics as a model species (Draper *et al.* 2001; Garvin *et al.* 2008; Mur *et al.* 2011; Brutnell *et al.* 2015). *B. distachyon* also has a number of advantages over the related domestic Pooideae for a GWAS approach as it is a wild species with a wide climatic distribution, resulting in diverse phenotypes, as well as wide genomic diversity, for traits involved in life strategy and abiotic stress tolerance. *B. distachyon* has a small, fully sequenced genome of 270 Mb (The International Brachypodium Initiative 2009) compared to the 16 Gb of wheat (The International Wheat Genome Sequencing Consortium 2017) or 5.1 Gb of barley (The International Brachypodium Initiative 2009). It also contains a low percentage of repetitive noncoding DNA at 21.4% of nucleotides compared to >80% in wheat (Wicker *et al.* 2011) and 84% in barley (The International Barley Genome Sequencing Consortium 2012). This means that sequence reads from *B. distachyon* are much easier to identify and align compared to wheat, with a larger proportion of the sequencing providing useful reads. Finally, and perhaps most importantly, the short stature of *B. distachyon* allows large numbers of plants to be taken through full life cycles in controlled growth conditions.

*Brachypodium* is widespread throughout temperate regions, including its native Mediterranean range and introduced range in Australia, South Africa, and the western United States (Vogel *et al.* 2009; Wilson and Jones 2015). A large number of accessions have been collected throughout the world by the *Brachypodium* community, but the use of these collections in genomic association studies has been delayed by the cryptic nature of the *Brachypodium* species complex. The three species in this complex are difficult to distinguish in the field and include the diploid *B. distachyon*, the diploid *B. stacei*, and the allotetraploid *B. hybridum*, which contains one *B. distachyon*-like genome and one *B. stacei*-like genome (Hasterok *et al.* 2004; Catalán *et al.* 2012; Idziak *et al.* 2014). To add to the complexity, there is evidence of distinct subgroups of *B. distachyon* (Hasterok *et al.* 2004; Catalán *et al.* 2012; Idziak *et al.* 2014; Tyler *et al.* 2016). While the genome of the Bd21 reference genotype of *B. distachyon* was published in 2010, the genome of *B. stacei* and other SNP corrected genomes were released online in 2016 (DOE-JGI, http://phytozome.jgi.doe.gov/). Recently, a *B. distachyon* pan genome was published identifying geographic diversity and many new genes not identified in the initial reference (Gordon *et al.* 2017). Prior to our study, species identification has commonly been undertaken by morphoanatomical classification, a small number of markers, or cytology (*e.g.*, Hasterok *et al.* 2004; The International Wheat Genome Sequencing Consortium 2017). There is a need for a rapid identification of species, subgroup, and genotype lineages within the *Brachypodium* species complex to aid the selection of HapMap sets, and to enable landscape genomic studies of migration and adaptation.

In this study, we aimed to (1) characterize the species, genotype, and population structure of a *Brachypodium* global diversity set to select a core haplotype mapping set for GWAS in *B. distachyon* and (2) identify the genetic architecture and plasticity of the agriculturally relevant traits of heading date, early vigor, and energy use efficiency in response to climate.

## Materials and Methods

### Genotyping by sequencing and species identification

Genotyping by sequencing (GBS) was undertaken as described by Elshire and colleagues (Elshire *et al.* 2011) using *Pst*I enzyme and a library of homemade barcoded adaptors (see https://github.com/borevitzlab/brachy-genotyping; Morris *et al*. 2011; Nicotra *et al*. 2016). Approximately 384 samples were multiplexed to run on a single lane in an Illumina HiSeq 2000 with a median number of 564,000 100-bp read pairs per sample (https://github.com/borevitzlab/brachy-genotyping). Sequencing runs were undertaken by the Biomolecular Resource Facility [The John Curton School of Medical Research (JCSMR), Australian National University (ANU)].

Axe (Murray and Borevitz 2018) was used to demultiplex sequencing lanes into libraries, allowing no mismatches. AdapterRemoval (Schubert *et al.* 2016) was used to remove contaminants from reads, and merge overlapping read pairs. Reads were aligned using BWA MEM (Li 2013; Li and Durbin 2009) to the Bd21-3 (*B. distachyon*) and ABR114 (*B. stacei*) reference genomes (Phytozome v.12.1), and to a *B. hybridum* pseudoreference genome created by concatenating the *B. stacei* and *B. distachyon* reference genomes (Supplemental Material, File S1). Variants were called using the multiallelic model of samtools mpileup (Li 2011) and bcftools call (Danecek *et al*. 2016). Variants were filtered with bcftools filter, keeping only SNPs of reasonable mapping and variant qualities (≥10) and sequencing depth across samples (≥5 reads across all samples).

To determine the species of each of the accessions, we computed the proportion of each chromosome in the *B. hybridum* pseudoreference covered with at least three reads, excluding reads that mapped to multiple locations in the pseudoreference, using mosdepth (Pedersen and Quinlan 2017). The proportions of the *B. distachyon/B. stacei* genomes covered were normalized to be in [0, 1], and then used to assign samples into threshold groups: *B. stacei* (<0.03), intermediate *B. stacei/B. hybridum* (<0.28), *B. hybridium* (<0.34), intermediate *B. hybridum/B. distachyon* (0.94), and *B. distachyon* (>0.94); an additional group consisted of low coverage samples (<100,000 reads in total). Samples from intermediate and low coverage groups were excluded, and only variants in the respective genomes were used to allocate the three species groups.

### Population structure of *B. distachyon*

To determine the population structure of *B. distachyon*, a pairwise identity-by-state (IBS) genetic distance was calculated to identify, among 490 high-quality samples, a core diversity set of 72 distinct genotypes using 82,800 SNPs derived from GBS data and the SNPRelate package using a z-score of 3.5. Occasionally, when genotypes are closely related, noise between technical replicates of an accession will result in them being split across the related genotypes. Therefore, we keep replicate(s) from the genotype with the majority of replicates for that accession, breaking ties by keeping the replicate with the lowest missing data. In addition, 29 accessions whose geographic origin was suspect were also excluded.

**Figure 1:**
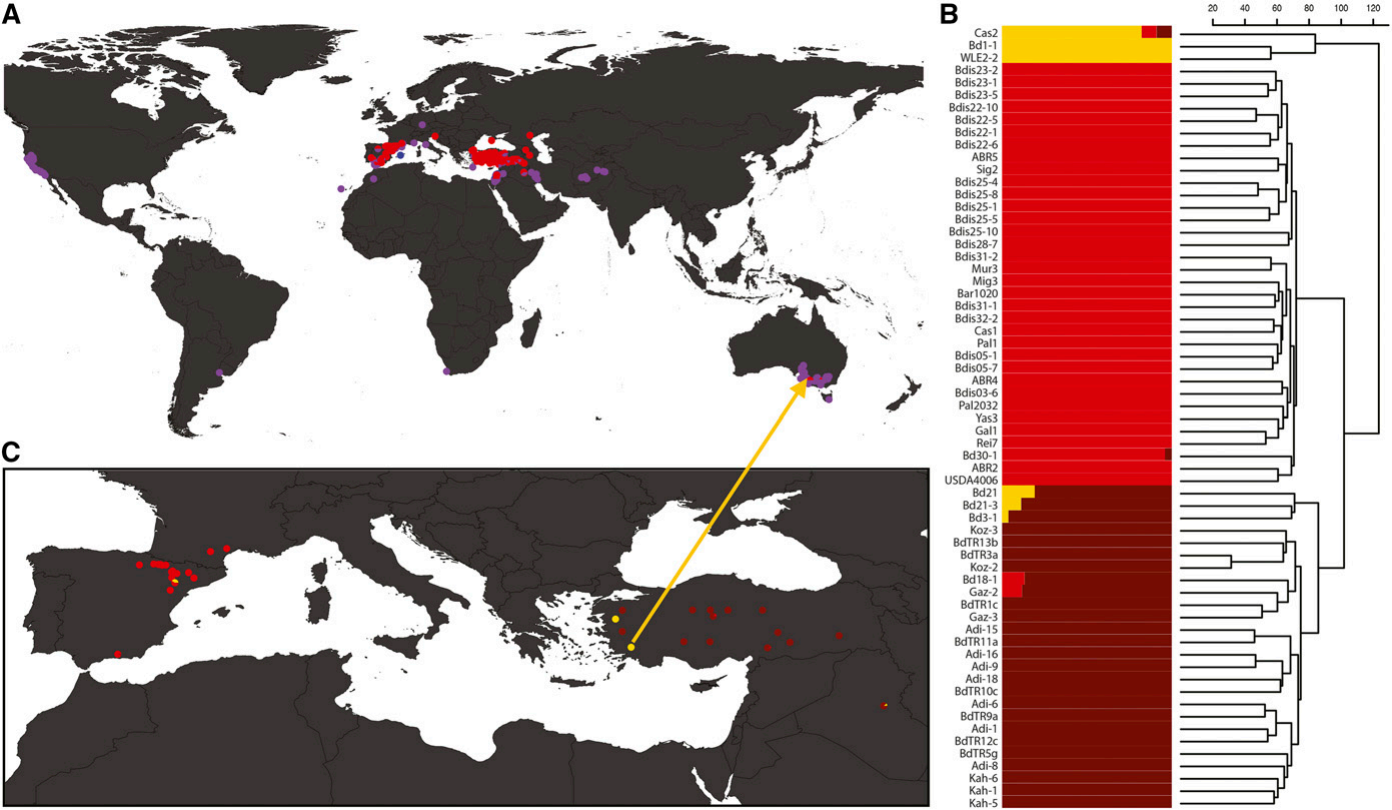
Distribution and genomic diversity of the *B. distachyon* complex. (A) Geographic distribution of 1858 Brachypodium complex accessions, classified by species: pink = *B. distacyhon*, blue = *B. stacei*, and purple = *B. hybridum*. (B) Population structure of the 63 diverse *B. distachyon* genotypes, *K* = 3. The three structure groups correspond to the B subgroup of *B. distachyon* (yellow), and the eastern (brown) and western (red) Mediterranean populations of the A subgroup of *B. distachyon*; and (C) geographic structure of *B. distachyon* across Iberian Peninsula and Turkish region. Proportions of pies represent the number of each *B. distachyon* subgroup (from B) at each site. The arrow from (C) to (A) shows the Australia *B. distachyon* (WLE2-2) and the near-identical accession from Turkey (BdTR9f).

To avoid bias from including up to 30 inbred accessions of the same genotype, a reduced set was input into STRUCTURE V.2.3.4 (Evanno *et al.* 2005). A total of six replicates were run of population (K) 1–13 with a burn-in setting of 10,000 sets, and 100,000 permutations per run (Figure 1B and File S3). The optimal K was determined as *K* = 3 by Evanno’s Delta K, processed via Structure Harvester and CLUMPP (Evanno *et al*. 2005, Jakobsson and Rosenberg 2007; Earl and vonHoldt 2012). Barplots and pie charts were generated via inhouse developed R scripts available through github (https://github.com/borevitzlab/brachy-genotyping-notes).

For *B. distachyon*, the pairwise distance between genotypes was also calculated in R and plotted as a dendrogram (File S2). From this, a set of 107 accessions were selected to represent the genotypic diversity of the species for whole genome sequencing (WGS) to maximize SNP coverage across the genome.

### Whole genome sequencing

For WGS, sequencing libraries for individual samples were prepared from 6 ng genomic DNA with the Nextera DNA Library Prep kit (Illumina, San Diego, CA). Libraries were enriched and barcoded with custom i5-, and i7-compatible oligos and Q5 High-Fidelity DNA Polymerase (NEB, Ipswich, MA). Libraries were pooled and sequenced in one lane on a NextSeq 500 sequencer (Illumina).

Trimit (Murray and Borevitz 2017) was used to clean WGS reads of adaptors, and merge overlapping read pairs. BWA MEM was then used to align these reads against the Bd21-1 reference genome (version 314_v3.1; The International Brachypodium Initiative 2009). Variants were called using freebayes (Garrison and Marth 2012) with default parameters. Variants were filtered such that only variants meeting the following criteria were kept: variant quality >20, minor allele frequency ≥2%. Heterozygous variant calls were changed to missing; due to the inbred nature of these accessions, heterozygous calls were almost certainly erroneous (https://github.com/borevitzlab/brachy-genotyping).

Linkage disequilibrium (LD) was calculated across the *B. distachyon* genome using consecutive windows of 2000 SNPs from the whole genome data of the HapMap 74 set (http://github.com/borevitzlab/brachy-genotyping-notes).

### Plant growth

Individual grain of each genotype was planted 2.5 cm deep in square plastic pots (5 cm width, 8 cm deep) in a mix of 50:50 soil:washed river sand that had been steam pasteurized. Pots were then placed at 4° in the dark for 3 days to stratify the seed before being moved to specially modified climate chambers (see Garrison and Marth 2012). Accessions were organized in a randomized block design, in trays of 20 plants. The chambers have been fitted with seven LED light panels and are controlled to change the light intensity, light spectrum, air temperature and humidity every 5 min. Seasonal changes in climatic conditions and photoperiod were modeled using SolarCalc software (Spokas and Forcella 2006). The Wagga Wagga region is centered on ∼ −35S, 147E with an elevation of 147 m. Plants were fertilized with Thrive (N:P:K 25:5:8.8 + trace elements, Yates) and watered with tap water as needed. Growth stages were recorded based on the Huan developmental stage (Haun *et al*. 1973) up until stem elongation and thereafter the Zadoks scale was used. Total leaf area was measured with a Li-1300 Area Meter (Li-COR). For dry weight, leaf tissue was dried in a paper envelope at 60° for 5 days before weighing.

### Conversions of phenotypic data

Thermal time was calculated from the logged condition within each chamber with the following formula:

If Temp._1>2°C,then TT_2=TT_1+[(Temp._2−2)×ΔTime]_(2−1)

where TT*_i_* is accumulated thermal time at a particular timepoint *i*, and Temp*._i_* is the air temperature at a particular timepoint *i*.

Photothermal units (PTU) were calculated using the logged data from a photosynthetically active radiation (PAR) sensor in the middle of the chamber and the following formula:

(PTU)_1=(TT)_1×(PAR)_1

where TT is the accumulated thermal time at timepoint *i* and PAR is the measured photosynthetically active radiation at timepoint *i*.

Growth rates (GR) were calculated as:

GR=[(ΔGS)_(T_2(−T)_1)]/[Δ(Time)_((T_2−T)_1)]

where GS is the Huan growth stage and T_1_ was about one leaf for the initial linear growth stage (GR1), T_1_ was about one leaf and three leaves, and the faster growth stage (GR2) between three leaves and five leaves. The Phyllachron interval, the time taken to grow one leaf, was calculated as:

Phyllachron Interval=(T_2−T)_e/(GS)_2

where T_2_ is the unit of time at about the three-leaf stage and T_e_ is the unit of time at seedling emergence for that particular plant. GS_2_ is the Huan growth stage at T_2_.

Final growth efficiency was calculated when plants reached ear emergence. The final growth efficiency 1 was calculated as:

Final growth efficiency1=(Biomass at ear emergence(g))/(ΔThermal time)

where accumulated thermal time is calculated from seedling emergence to ear emergence. The final growth efficiency 2 was calculated as:

Final growth efficiency2=(Biomass at ear emergence(g))/(ΔPhotothermal units)

where accumulated photothermal units is calculated from seedling emergence to ear emergence.

### Energy use efficiency traits

Energy use efficiency traits were measured on plants from the 2015–2050 Temperature experiment at a four- to five-leaf stage. Photosynthetic parameters were measured using a Trayscan system (PSI) incorporating pulse amplitude modification (PAM) chlorophyll fluorescence measures of quantum efficiency (Rungrat *et al*. 2016). The parameters measured included photosynthetic efficiency, nonphotochemical quenching and photo-inhibition. See File S2 for protocol.

Dark respiration rate was measured using the Q2 system (Astec Global) as in Scafaro *et al.* (2017). In brief, this system uses an oxygen-sensitive fluorescent dye embedded in a cap to monitor the oxygen depletion with a tube containing the sample. A 3 cm fragment in the center of the last fully expanded leaf of each plant was used to measure dark respiration per unit area and per unit dry mass.

Several energy use efficiency formulas were calculated. These included a ratio of dark respiration to photosynthesis, and measures of growth per unit dark respiration. These were as follows:

Energy use efficiency 1=1−(respiration per unit area)/(average photosynthetic efficiency)

Energy use efficiency 2=(seedling height)/(respiration per g dry weight)

Energy use efficiency 3=(leaf #3 length)/(respiration per g dry weight)

Energy use efficiency 4=(seedling height)/(respiration per unit area)

### Heritability

Broad-sense heritability was calculated from the phenotype data using the nlme package in R.

### GWAS analysis

In preparation for GWAS, the genotype data were filtered to remove nonvariant SNPs and redundant SNPs (*i.e.*, SNPs whose genotypes are not different from adjacent SNPs but have more missing data points). Then, SNPs with a minor allele frequency of <3% were filtered out. As there was 18.5% missing data in the original data set, imputation was undertaken. First, if the observed genotypes of two adjacent SNPs were not different, then the missing genotype of one SNP was replaced by the observed genotype of the other SNP. Second, the nearest neighbor (NN) method was implemented to impute the remaining missing genotypes based on Huang *et al.* (2010) with some modifications. The nearest 50 SNPs from each side of the SNP under imputation were selected to estimate similarity between each pair of accessions, and then the missing genotype of an accession was replaced by the observed majority genotype of the closest five accessions. These parameters were determined by simulations to achieve an optimal imputation success rate, which was 97.95% for our data. Finally, SNPs with a minor allele frequency <5% were filtered. For the phenotype data, the average value for the four replicates of each accession was calculated.

Linear mixed-effect models were employed to identify genetic variants underlying phenotypes of interest

y=xβ+zγ+u+ϵ

where ***y*** = (*y_1_*, *y_2_*, *……*., *y_n_*_)_^’^ denotes phenotypic values, ***x*** = (*x_ij_*)*_nx_*_(_*_k+_*_1)_ represents intercept and *k* covariates (if any) with effects **β**, ***z*** is a vector of the coded genotypes at a scanning locus with effect γ, ***u*** = (*u_1_*, *u_2_*, *……*, *u_n_*)^’^ represents polygenic variation, and ***ϵ*** = (*ϵ_1_*, *ϵ_2_*, *……*, *ϵ_n_*) the residual effect. It was assumed that ***u*** ∼ *N*(0, ***K***$\sigma_{g}^{2}$), ***ϵ*** ∼ *N*(0, *I*${}^{2}$) and ***u*** was independent of ***ϵ***. The genetic relationship matrix ***K*** was estimated by IBS from genotypic data with markers on the chromosome under scan being excluded to avoid proximal contamination (Listgarten *et al*. 2012; Cheng *et al*. 2013). Estimation of ***K*** and genome scan were performed in R package QTLRel (Cheng *et al*. 2011).

To determine a significance threshold, the permutation test was implemented on 1000 permutations of the phenotype data to estimate the genome-wide significance threshold at 0.05 for the trait of days to ear emergence. The significance threshold was determined to be a LOD (logarithm of odds) of 4.43583.

### Data availability

GBS and whole genome sequence data are available in the sequence read archive at NCBI, BioprojectID PRJNA505390. Supplemental Figures and tables are available in FigShare. Supplemental material available at Figshare: https://doi.org/10.25386/genetics.7345160.

## Results

### Cryptic *Brachypodium* species, diverse genotypes, and population structure identified using GBS

To establish a diverse set of germplasm, thousands of *Brachypodium* accessions were collected on trips to south-west Europe, south-eastern Australia, the western USA, and through collaborations with the international *Brachypodium* community (https://github.com/borevitzlab/brachy-genotyping/blob/master/metadata/brachy-metadata.csv). Out of these, 1968 accessions were grown to produce single-seed descent lines in the greenhouses at the ANU for subsequent genomic analysis. A reduced representation approach, *PstI* digest, GBS was used to genetically profile the accessions.

Although once described as a single species, *B. distachyon* has more recently been shown to exist as a species complex consisting of a 5 chromosome *B. distachyon*, 10 chromosome *B. stacei*, and a 15 chromosome allopolyploid *B. hybridum* (Catalán *et al.* 2012). To categorize each accession into species within the *Brachypodium* complex, GBS tags were mapped to a merged reference genome consisting of *B. distachyon* (Bd21-3) and *B. stacei* (ABR114) (v1.1 DOE-JG, https://phytozome.jgi.doe.gov). Most accessions were readily distinguished as having reads that aligned to either or both reference genomes (see *Materials and Methods*; File S1). The majority of accessions, 56% (1100/1968), were identified as *B. hybridum*. In contrast, only 3% (60/1968) were classified as *B. stacei*, while 35% (698/1968) were *B. distachyon*. The remaining 6% (110/1968) could not be definitively assigned. Mapping of the accessions’ geographic locations showed that *B. hybridum* has expanded across the globe, representing essentially all the collections outside the native range (Figure 1A). Conversely, *B. distachyon* is largely limited to the native Mediterranean and Western Asian regions, with *B. stacei* in the same area, but less common.

Due to the highly selfing nature of all *Brachypodium* species, we next sought to categorize accessions into unique whole genome genotypes representing a single inbred lineage. Of the 698 accessions identified as *B. distachyon*, 490 could be reliably genotyped at 81,400 SNPs. We used the SNPRelate package (Zheng *et al.* 2012) to cluster these 490 accessions into 72 genotypes (see *Materials and Methods*; https://github.com/borevitzlab/brachy-genotyping-notes; File S2). Recombinant inbred lines, included as positive controls, were often called as unique genotypes as expected, but were excluded from subsequent analysis of natural population structure.

### Whole genome variation

One or two accessions of each unique genotype was selected for further analysis. Whole genome sequencing was performed on this set of 107 *B. distachyon* accessions to determine high density variation at multiple levels, patterns of LD, and to enable GWAS. We identified 2,648,921 SNPs present in at least two accessions. Due to the high inbreeding and clonal family structure observed (File S4), we sought to select a representative accession from each inbred family, reducing 107 accessions to 63 highly diverse genotypes.

Previous genetic analysis on smaller data sets had shown *B. distachyon* to have substantial population structure, forming three groups representing ancestral structure in the Mediterranean region (Filiz *et al.* 2009; Vogel *et al.* 2009; Tyler *et al.* 2016; Gordon *et al.* 2017; Marques *et al.* 2017). To reduce data complexity, SNPs were subsampled to every 100th site to create a final SNP matrix of 26,490 variants that were fed into STRUCTURE v.2.3.4 (Pritchard *et al.* 2000; File S3). STRUCTURE analysis identified three main subgroups among *B. distachyon* genotypes and seven admixed lines (Figure 1B). The yellow lineage was the most diverged and represents subgroup B, with the brown and red structure groups representing the two populations of the A subgroup, split predominantly as an East and West population. Our STRUCTURE clustering is largely consistent with previous results on a smaller, partially overlapping sets of accessions (see Figure 4 of Tyler *et al*. 2016; Gordon *et al*. 2017). To visualize the geographic distribution, the ancestral group composition was summed across accessions for each geographic site (Figure 1C). The single *B. distachyon* accession from Australia, WLE2-2, was nearly identical to BdTR9f (GBS data, File S2) from southern western Turkey, from where it may have originated. It is shown in its ancestral location (Figure 1C, arrow).

Although there were only three accessions in the B subgroup, they diverged from the A subgroup with fixed differences at 6.5% of sites. By comparison, fixed divergence between the two clear subpopulations within the A subgroup was 1.5%. Finally, accessions within the same unique genotype diverged at between 0.1 and 0.4% of SNPs. A balanced set of representative accessions across the genotype lineages within just the A subgroup was selected for further genomic and phenomic analysis (File S4).

LD was calculated for consecutive windows of 2000 SNPs across the genome. There was large variation in LD, as the distance of decay to half maximal r2, across the genome (File S5) with the median LD 113 kb (50–235 kb interquartile range) and the maximum >2.4 Mb.

### Determining the traits and climatic conditions for GWAS in *B. distachyon*

For our GWAS study, we wanted to identify high-throughput nondestructive phenotypic measures with high heritability. We also wanted to determine the best environmental conditions to characterize our traits of interest. Hence, two preliminary experiments were undertaken, one for flowering time and one for early vigor.

Flowering time was chosen as an ideal trait for GWAS as it has high heritability in many species including Arabidopsis (Brachi *et al.* 2010) and barley (Maurer *et al.* 2015). Previous studies of *B. distachyon* revealed that the dependence of flowering time on vernalization and photoperiod varies between accessions (Higgins *et al.* 2010; Ream *et al.* 2014; Bettgenhaeuser *et al.* 2017; Woods *et al.* 2017). This study aimed to identify QTL for earliness *per se* in flowering, *i.e.*, those responsive to the accumulation of thermal time. Hence, a preliminary experiment was undertaken to determine if our conditions could meet the vernalization requirements of all *B. distachyon* accessions, and to determine which accessions had strong vernalization requirements in our conditions. To do this, 266 diverse A- and B-subgroup accessions, with five accessions replicated five to six times, were grown in both a simulated Winter sowing, starting June 1, and a Spring sowing, starting September 1, in Wagga Wagga, NSW, Australia (File S6). Ear emergence was monitored as a surrogate measure for flowering time, as flowering occurs largely within the ear in *B. distachyon* so is hard to accurately record (File S7). Out of the 266 accessions, there were 17 accessions that did not flower in the Spring condition, indicating a strong vernalization requirement (File S8A). All lines flowered in the Winter condition, indicating that night temperatures of 4° were sufficient to meet vernalization requirement. As expected, days to ear emergence showed a strong heritability in the Winter condition, as calculated from the replicated lines (*H*^2^ = 0.96). The thermal time to flowering was calculated to determine the dependence of flowering on the accumulation of thermal time. The fast cycling accessions, which did not require vernalization, still required a larger thermal time accumulation than the vernalization requiring accessions (File S8B). This indicates that these either have some low-level requirement for vernalization that is not being fully met in the Spring condition, or that the photoperiod is also a factor in this relationship. As this study aimed to identify QTL for earliness *per se* in flowering, *i.e.*, those responsive to the accumulation of thermal time, we attempted to exclude vernalization and photoperiod effects by focusing on the Winter condition for the GWAS experiment.

In temperate grass crops such as wheat and barley, early vigor can result in an increased yield in short seasons, or in seasons where there is high rainfall (reviewed in Wilson *et al*. 2015c). Often, the dimensions of seedling leaves are measured as a nondestructive surrogate measure for early vigor (Rebetzke and Richards 1999; Wilson *et al.* 2015b). To confirm that this was also an appropriate surrogate measure for early vigor in *B. distachyon*, a highly replicated (*n* = 10) validation experiment was performed on six diverse *B. distachyon* lines (File S9A) in a simulated Wagga Wagga, seasonal climate starting on September 1 (Spring). After 7 weeks, when plants had between four and five mainstem leaves, the dimensions of leaf #3, seedling height, total leaf area, and above-ground dry weight were measured and phenotypic correlations were calculated (File S9B). Broad sense heritability was also calculated to determine which early vigor trait would provide the most power for mapping QTL with GWAS (File S9C). Leaf #3 width and length correlated well with above-ground biomass (*r*^2^ = 0.46, *P* < 0.01, and *r*^2^ = 0.48, *P* < 0.01, respectively) and had quite high heritabilities of *H*^2^ = 0.60 and *H*^2^ = 0.64, respectively, as compared to above ground dry mass, *H*^2^ = 0.51. Interestingly, seedling height also had a strong correlation with above ground biomass (*r*^2^ = 0.74, *P* < 0.01) with a heritability of *H*^2^ = 0.74. However, this trait was also more highly correlated with developmental stage, as indicated by the number of leaves (*r*^2^ = 0.21, *P* < 0.01), than the dimensions of leaf #3. To get the most direct measure of early vigor, without the influence of developmental stage, the dimensions of leaf #3 were chosen as the focus for the GWAS.

### Selection of global HapMap set

High-level population structure confounds GWAS when there are few segregating SNPs in common between ancestral groups relative to variation within each subgroups (Brachi *et al.* 2011). Here, we focused on subgroup A, which contains a majority of unique genotypes, resulting in a HapMap set of 74 genotypes. Within the A subgroup there is still clear population structure, but further subset selection would limit both the sample size and the phenotypic and genotypic diversity, reducing the rate of true positive results. This residual relatedness between lines was accounted for by including a kinship matrix in the GWAS model.

### Early vigor and ear emergence show genotypic variation in response to different simulated environments

**Figure 2:**
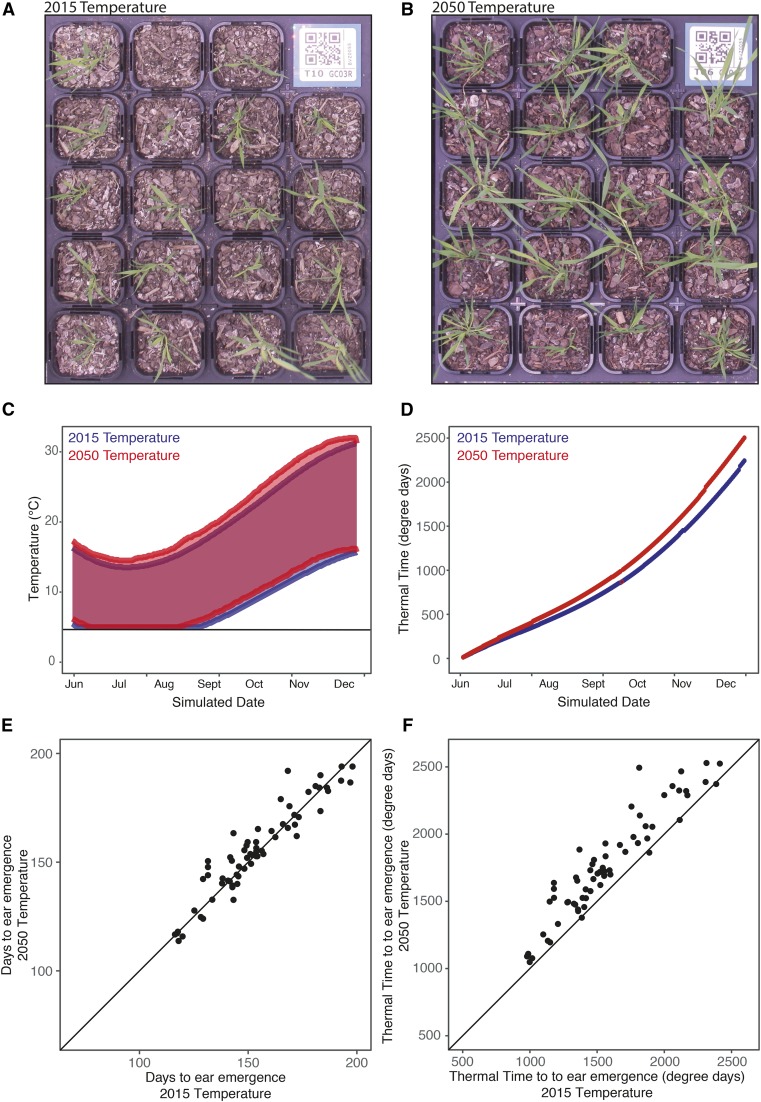
Differential growth of *B. distachyon* under current and future climate growth temperatures. Climate chambers were used to compare the response of agronomic traits to small change in the climate for a Winter sowing in the Wagga Wagga region, south-eastern Australia. The GWAS HapMap set were grown in (A) 2015 temperature climate and (B) a 2050 temperature climate. Photos show representative plants after 16 weeks of growth. Climate chambers were programmed to have (C) diurnal and seasonal changes in temperature resulting in different rates of accumulation of thermal time (D) in the 2015 and 2050 climates. Timing of ear emergence was compared between chambers for both (E) days to ear emergence and (F) the accumulation of thermal time to ear emergence, demonstrating G × E interactions.

To determine the genetic architecture for ear emergence date, early vigor, and a range of other agronomic traits (see *Materials and Methods*), the refined and balanced HapMap set of 74 *B. distachyon* accessions (File S10), with four biological replicates, were grown in two simulated conditions in climate chambers (Brown *et al*. 2014). To determine the effect of an increase in temperature in line with climate change predictions on the traits of interest, the conditions modeled a present (2015, Figure 2A) and a future (2050, Figure 2B) temperature profile at Wagga Wagga, NSW, Australia. The appropriate increase in average maximum and minimum temperature for each month were determined using an average of 12 global climate change models determined to be high confidence for south east Australia using the Climate Futures Tool (Figure 2C; Wilson *et al.* 2015a; File S11).

As expected, the accessions developed quicker and grew larger in the 2050 temperature profile (Figure 2, A and B) as is consistent with a quicker accumulation of thermal time (Figure 2D). Early vigor parameters and energy use efficiency traits were measured when the majority of plants were at a four-leaf stage. Growth stages, tiller numbers and ear emergence dates were monitored twice a week (File S12–S14). The experiment ceased after 200 days of growth, at which time there were five and seven lines that did not flower in the 2015 condition and 2050 conditions, respectively. The remaining lines reached ear emergence at a similar number of days in both the present and future conditions (Figure 2E). However, when converted to thermal time, those lines in the 2015 temperature condition required less thermal time than those in the 2050 temperature condition (Figure 2, D and F). This indicates that there is generally more dependence on photoperiod in this population than on thermal time to trigger the transition to flowering. There was variation between genotypes in the plasticity of their response to the two conditions (Figure 2, E and F), indicating that it would be worthwhile mapping the genotype by environment interaction (G × E).

### Determining the genetic architecture of early growth, ear emergence, and energy use efficiency traits in response to environment

**Figure 3:**
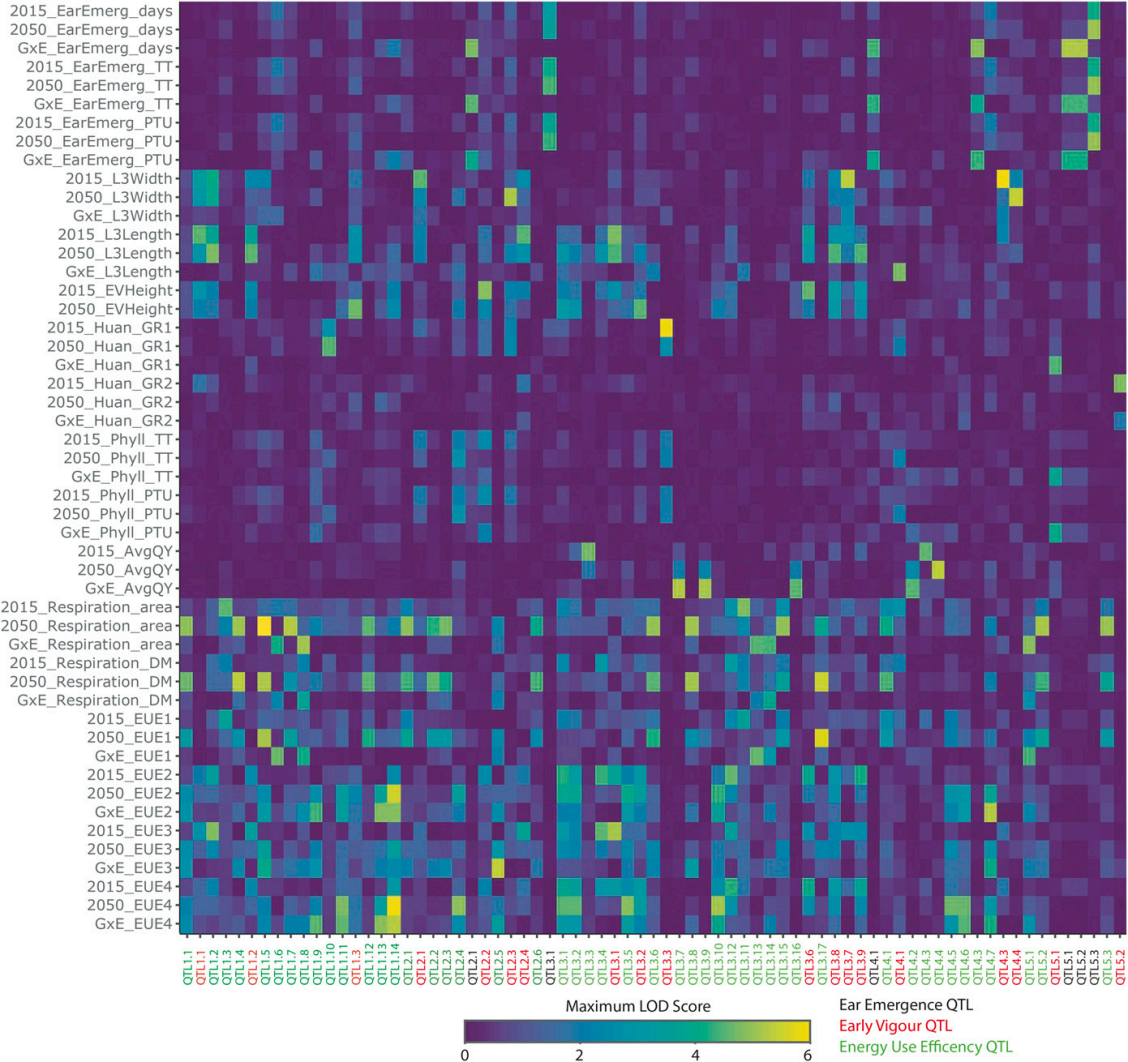
Summary of QTL for each trait under current and future climate growth temperatures. A total of 73 significant QTL were identified for a range of agronomic traits phenotyped in the 2015 temperature and 2050 temperature climates and the G × E interaction. There was little overlap between QTL for different traits but two robust QTL were identified in both environments while 16 QTL were identified for a G × E interaction. G × E, genotype by environment interaction; EarEmerg, ear emergence; TT, thermal time; PTU, photothermal units; L3Width, leaf 3 width; L3Length, leaf 3 length; GR, growth rate; GR, growth rate; EV, early vigor; phyll, phyllacron interval; AvgQY, average quantum yield; DM, dry mass; EUE, energy use efficiency

**Figure 4:**
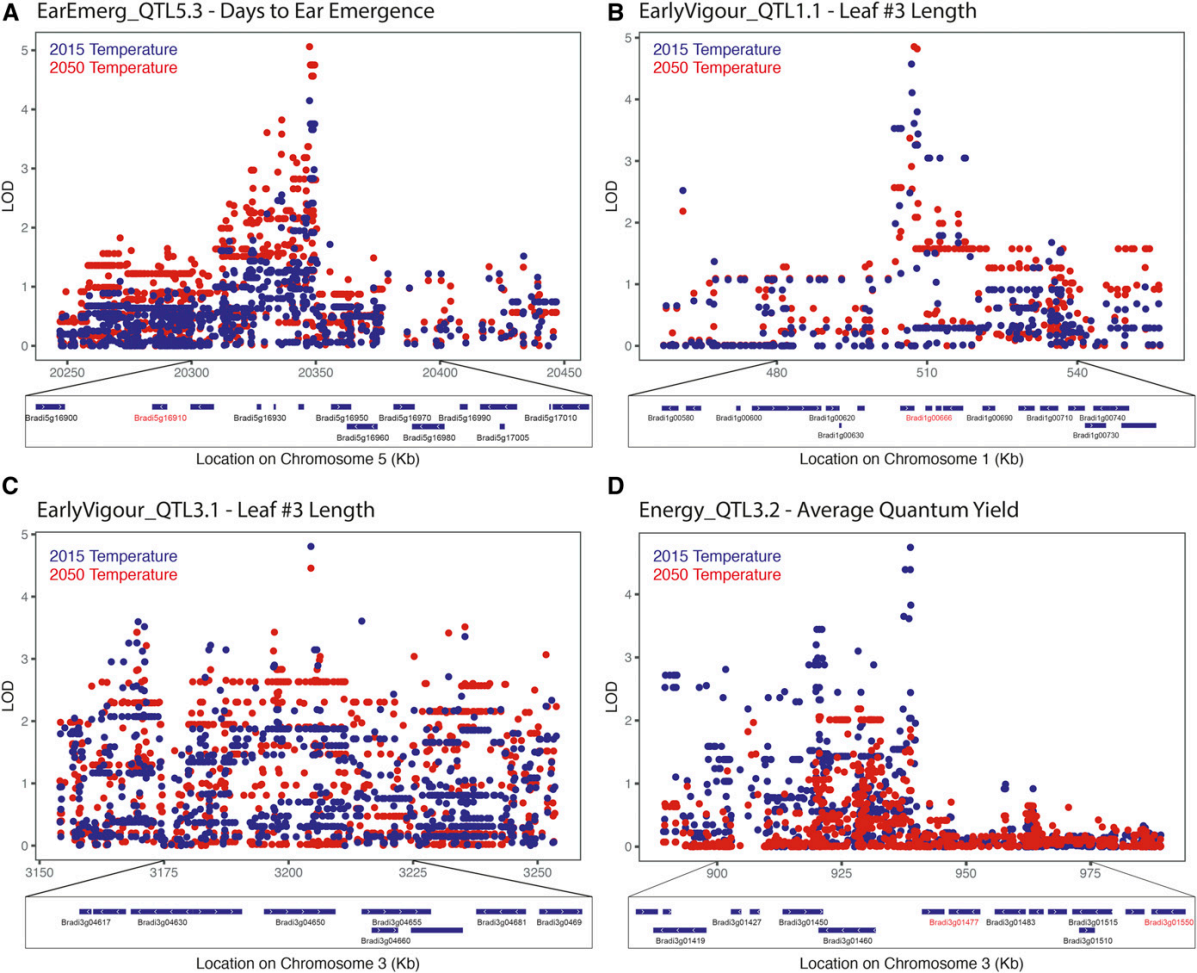
Putative candidate genes for QTL of key interest. (A) The ear emergence QTL, EarEmerg_QTL5.3, was significant for days to ear emergence in the 2050 temperature condition and only just under the significance threshold for the 2015 condition. Likely candidate genes include a YABBY transcription factor Bradi5g16910. (B) The early vigor QTL, EarlyVigour_QTL1.1 for leaf #3 length was found to be significant in both conditions. This region contains an ethylene sensitive transcription factor, Bradi1g00666. (C) The early vigor QTL, EarlyVigour_QTL3.1 was also identified for leaf #3 length in both environments. (D) A strong QTL was identified for photosynthetic efficiency, Energy_QTL3.2, which was significant only in the 2015 temperature condition. Likely candidate genes include a heat shock protein, Bradi3g01477, and a Low PSII Accumulation 3 (LPA3) protein, Bradi3g01550. Locus identifiers in red represent these candidate genes.

GWAS were performed on raw and derived traits as described in the *Materials and Methods* (Figure 3 and File S15 and File S16). All GWAS data are publicly available and traits are genome browseable online at https://easygwas.ethz.ch/gwas/myhistory/public/17/. For ear emergence, eight significant QTL were identified. EarEmerg_QTL4.2 explains 62% of the phenotypic variation in thermal time to ear emergence in the 2015 temperature condition, while two QTL, EarEmerg_QTL3.1 and EarEmerg_QTL5.3, explain 56 and 10%, respectively, of the phenotypic variance in thermal time to ear emergence in the 2050 temperature condition. No QTL were found to be significant in both conditions but EarEmerg_QTL5.3 was significant in the 2050 temperature condition and was just under the significant threshold in the 2015 temperature condition (Figure 4A and File S17). Within the 100 kb region of this SNP there are 15 genes, several of which could be relevant to the regulation of flowering, including a YABBY transcription factor (Bradi5g16910), a no apical meristem (NAM) protein (Bradi5g16917), and an expressed gene containing a RNA recognition motif (Bradi5g16930). Interestingly, there were two QTL that were significant for thermal time to ear emergence, EarEmerg_QTL3.1 and EarEmerg_QTL4.2, but not for days to ear emergence. There were six QTL identified for the G × E interaction, explaining, in part, the variation among lines in response to future climate.

For early vigor, 22 significant QTL were identified for five traits across the two climate conditions (File S15). Two QTL were identified in both conditions, EarlyVigour_QTL1.1 and EarlyVigour_QTL3.1, and both of these were for leaf #3 length. The 100-kb region surrounding these QTL contained 19 and 13 genes, respectively (Figure 4, B and C). There was a highly significant QTL on chromosome three for growth rate 1, EarlyVigour_QTL3.3, a measure of the rate of development of the seedling at the two leaf stage, but only in the 2015 temperature condition. The 100-kb region surrounding this QTL contained 13 genes (File S18). A total of six QTL were identified for the G × E interaction across the two conditions for early vigor traits.

For the energy use efficiency traits, a total of 47 QTL were identified across the two conditions for the three measured traits and four derived traits (File S15). Of these QTL, none were found in both environments. However, a strong QTL, Energy_QTL3.3, was identified for average quantum yield, a measure of photosynthetic efficiency, in the 2015 temperature environment. The 100-kb region around this QTL contained 24 genes including a low PSII accumulation three chloroplastic protein (Bradi3g01550), a Heat Shock Protein (Bradi3g01477) and several transcription factors (Figure 4D and File S19).

## Discussion

Thanks to the international *Brachypodium* community, in addition to our own collections, here we were able to provide the most comprehensive survey of *Brachypodium* species complex diversity to date. With 1968 accessions across the globe this is a >10-fold increase from previous studies (Filiz *et al.* 2009; Tyler *et al.* 2016).

Since being described as three separate species in 2012 (Catalán *et al.* 2012), species identification in the *Brachypodium* species complex has been achieved by morphology, PCR of a select set of markers or DNA barcoding (*e.g.*, Rebetzke *et al.* 1999a; Wilson *et al.* 2015b). Here, we present a unique systematic method of determining the species of an accession using low coverage GBS and bioinformatics, providing a high-throughput and low-cost alternative for species identification. We found that the majority of our accessions were *B. hybridum* (56%), including the vast majority of accessions in Australia and North America (Figure 1A). The wide dispersion of this species may be due to the benefit of the multiple genomes resulting from polyploidization (te Beest *et al.* 2012). There were relatively few *B. stacei* (3%), which were limited to the Mediterranean region (Figure 1A).

Within *B. distachyon* itself, we found significant population structure, including high level subgroup splits, with 6.5% of SNPs diverged between subgroups, which is greater than that found between *indica* and *japonica* rice at 1.4% divergence (Ma and Bennetzen 2004). While many previous studies have focused on individual regions (Filiz *et al.* 2009; Marques *et al.* 2017), the collection of 490 diverse *B. distachyon* accessions genotyped at 81,400 high quality SNPs presented here has allowed us to further distinguish groups with the *B. distachyon* subgroups, with an eastern and western European group in each subgroup. A number of geographically diverse highly related genotypic lineages were also identified, which showed within-lineage divergence of between 0.1 and 0.4% of SNPs. The geographic spread of these lineages highlights the inbreeding nature and high dispersal ability of *B. distachyon*.

The hierarchical levels of genetic variation within the *Brachypodium* species complex can be attributed to allopolyploidization and subspeciation, possibly during the most recent ice age; east/west IBD in Europe; and the high levels of self-fertilization in the species (Wilson *et al.* 2015a). These levels of population structure have been seen in Arabidopsis (Atwell *et al.* 2010), and other highly selfing plant species such as barley (Wang *et al.* 2012), but are more extreme in *Brachypodium*. In rice, either the *indica* sub-species (Huang *et al.* 2012) or *japonica* subspecies (Yano *et al.* 2016), have been separately used for GWAS. Similarly, to deal with the population structure in this study, the HapMap set was limited to the A subgroup of *B. distachyon* with remaining relatedness included in the GWAS analysis using mixed models (Cheng *et al.* 2011).

The lack of recombinant genetic diversity with subgroup and populations of *B. distachyon* also limits the power of GWAS analysis. The HapMap set contains a large amount of genomic diversity (>1% of bases are variable) but the sample size is low and the extent of LD is high, limiting mapping resolution. However, the patterns are similar to rice where GWAS is very effective as sample size increases (Huang and Han 2014). The construction of a Nested Association Mapping (NAM) population for *B. distachyon* would be advantageous to break-up the population and familial lineages, and to increase the frequency of minor alleles. This has been a successful approach in other species such as maize and wheat (Tian *et al.* 2011; Bajgain *et al.* 2016). Nevertheless, our set of lines and genomic data available in easyGWAS are an important resource for the community to map the genetic basis of various complex traits in this emerging model grass species. The small stature and rapid generation time of Brachypodium makes it especially useful for high throughput assays of phenomic traits across a range of controlled conditions.

In field conditions, determining the relationship between various physiological traits and their impact on yield is difficult due to seasonal environmental variability and the presence of a range of abiotic and biotic stresses. However, experiments in growth chambers often have little relevance to field conditions due to the unrealistic and static nature of the conditions. By using dynamic growth conditions, which mimic regional climates, we can avoid the stochastic downsides of field experiments while providing results arguably more translatable to the field (Brown *et al.* 2014; Poorter *et al.* 2016). The use of climate chambers also allows the impact of small changes in climate to be observed, and the dissection of which components of the climate have the largest influence on a trait of interest. In this study, we examined the effect of an increase in temperature in line with climate change model predictions for 2050 in south eastern Australia. Unexpectedly, there was generally a short delay of flowering time in the 2050 temperature condition, with variation in the extent of delay in different genotypes, while there was little dependence of flowering on the accumulation of thermal time. This suggests that there may be some vernalization requirements in *B. distachyon* that are not being met in the 2050 temperature condition. The lack of vernalization is also evident in the fact that seven lines had not flowered by the end of the 2050 temperature condition, while five lines did not flower in the 2015 temperature condition. While this GWAS analysis did not identify known flowering time loci that regulate vernalization-induced flowering such as VRN1, VRN2, and FT (Woods *et al.* 2014; Bettgenhaeuser *et al.* 2017), the QTL may represent more subtle vernalization processes that would be important for facultative varieties. Perhaps largely to the difference in growth conditions, the QTL in this study did not overlap with those found in a previous GWAS of flowering time (Tyler *et al.* 2016); this may also be an example of the Beavis effect (Xu 2003). Candidate genes identified for flowering time here included several transcription factors, including a YABBY transcription factor under EarEmerg_QTL5.3. The closest rice ortholog, Os04g45330, to this YABBY transcription factor is most highly expressed in the shoot apical meristem and developing inflorescence (Rice Gene Expression Atlas), while the closest Arabidopsis ortholog, At2g45190, is involved in regulation of the floral morphology (Lu *et al.* 2007). This EarEmerg_QTL5.3 was significant in the 2050 temperature conditions and was only just below the significance threshold in the 2015 temperature condition (Figure 4A).

Early vigor is an important trait in many parts of Australia and the rest of the world, where there is competition from weeds and a shorter season. Despite the highest correlating nondestructive measure of early above ground biomass being seedling height, the most robust QTL across environments were actually identified by leaf #3 length. Two QTL identified for leaf #3 length were identified in both environments, indicating they could potentially be useful for breeding for early vigor in multiple environment types. One of these, EarlyVigour_QTL1.1 is located in an area of synteny to other areas where early vigor QTL have been identified at the end of chromosome 3 in rice (Lu *et al*. 2007; The International Brachypodium Initiative 2009; Singh *et al*. 2017) and Chromosome 4 in wheat (Rebetzke *et al.* 2001). Within EarlyVigour_QTL1.1 there is a candidate gene, Bradi1g00666, that is described as an ethylene-responsive transcription factor. The main candidate gene in the QTL on chromosome 3 in rice was also an ethylene responsive gene (Singh *et al.* 2017). The EarlyVigour_QTL3.1 for leaf #3 length was also found to be significant across both environments. There were no obvious candidate genes for this QTL, but a number of signaling proteins that could be involved in molecular control of leaf size (Figure 4C and File S18).

The balance of energy production and use in plants is highly linked to the conditions that the plant is grown under; however, genetic variation controlling the energy efficiency of plants could be used to increase yield potentials. The quantum yield is an indicator of photosynthetic efficiency, the proportion of energy harvested through the light-harvesting complexes that goes toward producing photosynthates (Rungrat *et al.* 2016). No QTL were identified in common across both environments, but there were 11 QTL that were identified for the G × E interaction. This may be due to the sensitivity of these energy processes to the subtle difference in environments or a result of being measured on different days to allow comparison of plants at the same developmental stage. A strong QTL was identified for quantum yield, a measure of the efficiency of Photosystem II (PSII), in the 2015 climate but, interestingly, not in the 2050 climate. Candidate genes under this QTL included a gene with 66% homology to the Low PSII Accumulation 3 (LPA3) gene in Arabidopsis, which has been shown to be important in PSII assembly (Lu 2016). Further studies into the importance of this QTL in different conditions, as well as the other photosynthesis and respiration QTL, would be worthwhile.

In conclusion, the *Brachypodium* species complex is heavily structured at the ploidy, subgroups, population, and family levels. This limits the ability to identify the genetic basis of adaptation as relatively few recombinant genotypes were obtained. Despite these limitations, this study indicates the potential to use *Brachypodium distachyon*, a model for Pooideae grass crops, to identify genetic variation in key pathways underlying agricultural traits through GWAS. Further wild collections and/or the development of NAM populations could address the limitation of recombinant genotypes and result in very high power mapping population typical of 1000 genome projects. As it now stands, *Brachypodium* is a good model for both polyploidization, with likely multiple events among small divergent genomes, and for invasion biology with multiple widespread genotypes identified across continents, regions, and sites.

## References

[bib1] Atwell S. Huang Y. S. Vilhjalmsson B. J. Willems G. Horton M., 2010 Genome-wide association study of 107 phenotypes in Arabidopsis thaliana inbred lines. Nature 465: 627–631. 10.1038/nature08800 20336072 PMC3023908

[bib2] Bajgain P. Rouse M. N. Tsilo T. J. Macharia G. K. Bhavani S., 2016 Nested association mapping of stem rust resistance in wheat using genotyping by sequencing. PLoS One 11: e0155760 10.1371/journal.pone.0155760 27186883 PMC4870046

[bib3] Bettgenhaeuser J. Corke F. M. K. Opanowicz M. Green P. Hernández-Pinzón I., 2017 Natural variation in *Brachypodium* links vernalization and flowering time loci as major flowering determinants. Plant Physiol. 173: 256–268. Available at: http://www.plantphysiol.org/lookup/doi/10.1104/pp.16.00813. 10.1104/pp.16.00813 27650449 PMC5210709

[bib5] Brachi B. Faure N. Horton M. Flahauw E. Vazquez A., 2010 Linkage and association mapping of Arabidopsis thaliana flowering time in nature. PLoS Genet. 6: e1000940 10.1371/journal.pgen.1000940 20463887 PMC2865524

[bib6] Brachi B. Morris G. P. Borevitz J. O., 2011 Genome-wide association studies in plants: the missing heritability is in the field. Genome Biol. 12: 232 10.1186/gb-2011-12-10-232 22035733 PMC3333769

[bib7] Brown T. B. Cheng R. Sirault X. R. R. Rungrat T. Murray K. D., 2014 TraitCapture: genomic and environment modelling of plant phenomic data. Curr. Opin. Plant Biol. 18: 73–79. Available at: http://www.sciencedirect.com/science/article/pii/S1369526614000181. 10.1016/j.pbi.2014.02.002 24646691

[bib8] Brutnell T. P. Bennetzen J. L. Vogel J. P., 2015 *Brachypodium* distachyon and Setaria viridis: model genetic systems for the grasses. Annu. Rev. Plant Biol. 66: 465–485. 10.1146/annurev-arplant-042811-105528 25621515

[bib9] Catalán P. Muller J. Hasterok R. Jenkins G. Mur L. A. J., 2012 Evolution and taxonomic split of the model grass *Brachypodium* distachyon. Ann. Bot. 109: 385–405. 10.1093/aob/mcr294 22213013 PMC3268539

[bib10] Catalán P. Lopez-Alvarez D. Bellosta C. Villar L., 2016 Updated taxonomic descriptions, iconography, and habitat preferences of *Brachypodium* distachyon, B. stacei, and B. hybridum (Poaceae). An del Jard Bot Madrid. 73: e028 10.3989/ajbm.2428

[bib11] Cheng R. Abney M. Palmer A. A. Skol A. D., 2011 QTLRel: an R package for genome-wide association studies in which relatedness is a concern. BMC Genet. 12: 66 10.1186/1471-2156-12-66 21794153 PMC3160955

[bib13] Climate Change in Australia website, 2015 Available at: https://www.climatechangeinaustralia.gov.au/en/climate-projections/climate-futures-tool/introduction-climate-futures/. Accessed: September 1, 2015

[bib14] Condon A. G. Richards R. A. Rebetzke G. J. Farquhar G. D., 2004 Breeding for high water-use efficiency. J. Exp. Bot. 55: 2447–2460. 10.1093/jxb/erh277 15475373

[bib15] Danecek P. Schiffels S. Durbin R., 2016 Multiallelic calling model in bcftools (-m). Available at: http://samtools.github.io/bcftools/call-m.pdf.

[bib16] Draper J. Mur L. A. J. Jenkins G. Ghosh-Biswas G. C. Bablak P., 2001 *Brachypodium* distachyon. A new model system for functional genomics in grasses. Plant Physiol. 127: 1539–1555. Available at: http://www.plantphysiol.org/content/127/4/1539. 10.1104/pp.010196 11743099 PMC133562

[bib17] Earl D. A. vonHoldt B. M., 2012 STRUCTURE HARVESTER: a website and program for visualizing STRUCTURE output and implementing the Evanno method. Conserv. Genet. Resour. 4: 359–361. 10.1007/s12686-011-9548-7

[bib18] Elshire R. J. Glaubitz J. C. Sun Q. Poland J. A. Kawamoto K., 2011 A robust, simple genotyping-by-sequencing (GBS) approach for high diversity species. PLoS One 6: e19379 10.1371/journal.pone.0019379 21573248 PMC3087801

[bib19] Evanno G. Regnaut S. Goudet J., 2005 Detecting the number of clusters of individuals using the software STRUCTURE: a simulation study. Mol. Ecol. 14: 2611–2620. 10.1111/j.1365-294X.2005.02553.x 15969739

[bib20] Filiz E. Ozdemir B. S. Budak F. Vogel J. P. Tuna M., 2009 Molecular, morphological, and cytological analysis of diverse *Brachypodium* distachyon inbred lines. Genome 52: 876–890. 10.1139/G09-062 19935911

[bib21] Garrison E. Marth G., 2012 Haplotype-based variant detection from short-read sequencing. arXiv:1207.3907v2 [q-bio.GN]

[bib22] Garvin D. F. Gu Y. Q. Hasterok R. Hazen S. P. Jenkins G., 2008 Development of genetic and genomic research resources for *Brachypodium* distachyon, a new model system for grass crop research. Crop Sci. 48: 69–84.

[bib23] Giraldo P. Rodríguez-Quijano M. Vázquez J. F. Carrillo J. M. Benavente E., 2012 Validation of microsatellite markers for cytotype discrimination in the model grass *Brachypodium* distachyon. Genome 55: 523–527. 10.1139/g2012-039 22788413

[bib24] Gordon S. P. Contreras-Moreira B. Woods D. P. Vogel J. P., 2017 Extensive gene content variation in the *Brachypodium* distachyon pan-genome correlates with population structure. Nat. Commun. 8: 2184 10.1038/s41467-017-02292-8 29259172 PMC5736591

[bib25] Hasterok R. Draper J. Jenkins G., 2004 Laying the cytotaxonomic foundations of a new model grass, *Brachypodium* distachyon (L.) beauv. Chromosome Res. 12: 397–403. 10.1023/B:CHRO.0000034130.35983.99 15241018

[bib26] Haun J. R., 1973 Visual quantification of wheat development. Agron. J. 65: 116–119. 10.2134/agronj1973.00021962006500010035x

[bib27] Higgins J. A. Bailey P. C. Laurie D. A., 2010 Comparative genomics of flowering time pathways using *Brachypodium* distachyon as a model for the temperate grasses. PLoS One 5: e10065 10.1371/journal.pone.0010065 20419097 PMC2856676

[bib28] Huang X. Han B., 2014 Natural variations and genome-wide association studies in crop plants. Annu. Rev. Plant Biol. 65: 531–551. 10.1146/annurev-arplant-050213-035715 24274033

[bib29] Huang X. Wei X. Sang T. Zhao Q. Feng Q., 2010 Genome-wide association studies of 14 agronomic traits in rice landraces. Nat. Genet. 42: 961–967. 10.1038/ng.695 20972439

[bib30] Huang X. Zhao Y. Wei X. Li C. Wang A., 2012 Genome-wide association study of flowering time and grain yield traits in a worldwide collection of rice germplasm. Nat. Genet. 44: 32–39. 10.1038/ng.1018 22138690

[bib31] Idziak D. Hazuka I. Poliwczak B. Wiszynska A. Wolny E., 2014 Insight into the karyotype evolution of *Brachypodium* species using comparative chromosome barcoding. PLoS One 9: e93503 10.1371/journal.pone.0093503 24675822 PMC3968144

[bib32] Jakobsson M. Rosenberg N. A., 2007 CLUMPP: a cluster matching and permutation program for dealing with label switching and multimodality in analysis of population structure. Bioinformatics 23: 1801–1806. 10.1093/bioinformatics/btm233 17485429

[bib33] Li H., 2011 A statistical framework for SNP calling, mutation discovery, association mapping and population genetical parameter estimation from sequencing data. Bioinformatics 27: 2987–2993. 10.1093/bioinformatics/btr509 21903627 PMC3198575

[bib34] Li H., 2013 Aligning sequence reads, clone sequences and assembly contigs with BWA-MEM. arXiv:1303.3997v2 [q-bio.GN].

[bib35] Li H. Durbin R., 2009 Fast and accurate short read alignment with Burrows-Wheeler transform. Bioinformatics 25: 1754–1760. 10.1093/bioinformatics/btp324 19451168 PMC2705234

[bib36] Listgarten J. Lippert C. Kadie C. M. Davidson R. I. Eskin E., 2012 Improved linear mixed models for genome-wide association studies. Nat. Methods 9: 525–526. 10.1038/nmeth.2037 22669648 PMC3597090

[bib37] López-Alvarez D. López-Herranz M. L. Betekhtin A. Catalán P., 2012 A DNA barcoding method to discriminate between the model plant *Brachypodium distachyon* and its close relatives *B. stacei* and *B. hybridum* (Poaceae). PLoS One 7: e51058 Available at: http://www.pubmedcentral.nih.gov/articlerender.fcgi?artid=3519806&tool=pmcentrez&rendertype=abstract. 10.1371/journal.pone.0051058 23240000 PMC3519806

[bib38] Lu X. L. Niu A. L. Cai H. Y. Zhao Y. Liu J. W., 2007 Genetic dissection of seedling and early vigor in a recombinant inbred line population of rice. Plant Sci. 172: 212–220. 10.1016/j.plantsci.2006.08.012

[bib39] Lu Y., 2016 Identification and roles of photosystem II assembly, stability, and repair factors in Arabidopsis. Front. Plant Sci. 7: 168 Available at: http://journal.frontiersin.org/Article/10.3389/fpls.2016.00168/abstract. 10.3389/fpls.2016.00168 26909098 PMC4754418

[bib40] Ma J. Bennetzen J. L., 2004 Rapid recent growth and divergence of rice nuclear genomes. Proc. Natl. Acad. Sci. USA 101: 12404–12410. Available at: http://www.pnas.org/cgi/doi/10.1073/pnas.0403715101. 10.1073/pnas.0403715101 15240870 PMC515075

[bib41] Marques I. Shiposha V. López-Alvarez D. Manzaneda A. J. Hernandez P., 2017 Environmental isolation explains Iberian genetic diversity in the highly homozygous model grass *Brachypodium* distachyon. BMC Evol. Biol. 17: 139 10.1186/s12862-017-0996-x 28619047 PMC5472904

[bib42] Maurer A. Draba V. Jiang Y. Schnaithmann F. Sharma R., 2015 Modelling the genetic architecture of flowering time control in barley through nested association mapping. BMC Genomics 16: 290 Available at: http://www.biomedcentral.com/1471–2164/16/290. 10.1186/s12864-015-1459-7 25887319 PMC4426605

[bib43] Morris G. P. Grabowski P. P. Borevitz J. O., 2011 Genomic diversity in switchgrass (*Panicum virgatum*): from the continental scale to a dune landscape. Mol. Ecol. 20: 4938–4952. 10.1111/j.1365-294X.2011.05335.x 22060816 PMC3383086

[bib44] Mur L. A. J. Allainguillaume J. Catalan P. Hasterok R. Jenkins G., 2011 Exploiting the *Brachypodium* tool box in cereal and grass research. New Phytol. 191: 334–347. 10.1111/j.1469-8137.2011.03748.x 21623796

[bib45] Murray K. D. Borevitz J. O., 2017 libqcpp: a C++14 sequence quality control library. J Open Source Softw. 2: 232 10.21105/joss.00232

[bib46] Murray K. D. Borevitz J. O., 2018 Axe: rapid, competitive sequence read demultiplexing using a trie. Bioinformatics 34: 3924–3925. 10.1093/bioinformatics/bty432 29868827

[bib47] Nicotra A. B. Chong C. Bragg J. G. Ong C. R. Aitken N. C., 2016 Population and phylogenomic decomposition via genotyping-by-sequencing in Australian Pelargonium. Mol. Ecol. 25: 2000–2014. 10.1111/mec.13584 26864117

[bib48] Pedersen, B. S., and A. R. Quinlan, 2017 Mosdepth: quick coverage calculation for genomes and exomes. Bioinformatics. Available at: http://academic.oup.com/bioinformatics/advance-article/doi/10.1093/bioinformatics/btx699/4583630. 10.1093/bioinformatics/btx699 PMC6030888 29096012

[bib49] Poorter H. Fiorani F. Pieruschka R. Wojciechowski T. van der Putten W. H., 2016 Pampered inside, pestered outside? Differences and similarities between plants growing in controlled conditions and in the field. New Phytol. 212: 838–855. 10.1111/nph.14243 27783423

[bib50] Pritchard J. K. Stephens M. Donnelly P., 2000 Inference of population structure using multilocus genotype data. Genetics 155: 945–959. 10835412 10.1093/genetics/155.2.945 PMC1461096

[bib51] Ray D. K. Mueller N. D. West P. C. Foley J. A., 2013 Yield trends are insufficient to double global crop production by 2050. PLoS One 8: e66428 10.1371/journal.pone.0066428 23840465 PMC3686737

[bib52] Ream T. S. Woods D. P. Schwartz C. J. Sanabria C. P. Mahoy J., 2014 Interaction of photoperiod and vernalization determines flowering time of *Brachypodium* distachyon. Plant Physiol. 164: 694–709. Available at: http://www.pubmedcentral.nih.gov/articlerender.fcgi?artid=3912099&tool=pmcentrez&rendertype=abstract. 10.1104/pp.113.232678 24357601 PMC3912099

[bib53] Rebetzke G. J. Richards R. A., 1999 Genetic improvement of early vigour in wheat. Aust. J. Agric. Res. 50: 291–301. 10.1071/A98125

[bib54] Rebetzke G. J. Appels R. Morrison A. D. Richards R. A. McDonald G., 2001 Quantitative trait loci on chromosome 4B for coleoptile length and early vigour in wheat (*Triticum aestivum* L.). Aust. J. Agric. Res. 52: 1221–1234. 10.1071/AR01042

[bib55] Rivers J. Warthmann N. Pogson B. J. Borevitz J. O., 2015 Genomic breeding for food, environment and livelihoods. Food Secur. 7: 375–382. 10.1007/s12571-015-0431-3

[bib56] Rungrat T. Awlia M. Brown T. Cheng R. Sirault X., 2016 Using phenomic analysis of photosynthetic function for abiotic stress response gene discovery. Arabidopsis Book 14: e0185. 27695390 10.1199/tab.0185 PMC5042155

[bib57] Scafaro A. P. Negrini A. C. A. O’Leary B. Rashid F. A. A. Hayes L., 2017 The combination of gas-phase fluorophore technology and automation to enable high-throughput analysis of plant respiration. Plant Methods 13: 16 10.1186/s13007-017-0169-3 28344635 PMC5361846

[bib58] Schubert M. Lindgreen S. Orlando L., 2016 AdapterRemoval v2: rapid adapter trimming, identification, and read merging. BMC Res. Notes 9: 88 10.1186/s13104-016-1900-2 26868221 PMC4751634

[bib59] Singh U. M. Yadav S. Dixit S. Ramayya P. J. Devi M. N., 2017 QTL hotspots for early vigor and related traits under dry direct-seeded system in rice (Oryza sativa L.). Front. Plant Sci. 8: 286 Available at: https://www.frontiersin.org/article/10.3389/fpls.2017.00286. 10.3389/fpls.2017.00286 28303149 PMC5332406

[bib60] Spokas K. Forcella F., 2006 Estimating hourly incoming solar radiation from limited meteorological data. Weed Sci. 54: 182–189. Available at: https://www.cambridge.org/core/product/identifier/S0043174500007670/type/journal_article. 10.1614/WS-05-098R.1

[bib61] Sukumaran S. Dreisigacker S. Lopes M. Chavez P. Reynolds M. P., 2014 Genome-wide association study for grain yield and related traits in an elite spring wheat population grown in temperate irrigated environments. Theor. Appl. Genet. 128: 353–363. 10.1007/s00122-014-2435-3 25490985

[bib62] te Beest M. Le Roux J. J. Richardson D. M. Brysting A. K. Suda J., 2012 The more the better? The role of polyploidy in facilitating plant invasions. Ann. Bot. 109: 19–45. 10.1093/aob/mcr277 22040744 PMC3241594

[bib63] The International Barley Genome Sequencing Consortium, 2012 A physical, genetic and functional sequence assembly of the barley genome. Nature 491: 711–716. 10.1038/nature11543 23075845

[bib64] The International Brachypodium Initiative, 2009 Genome sequencing and analysis of the model grass Brachypodium distachyon. Nature 463: 763–768. Available at: http://link.springer.com/10.1007/s11103–009–9456–3. 10.1038/nature08747 20148030

[bib65] The International Wheat Genome Sequencing Consortium, 2017 Available at: www.wheatgenome.org.

[bib66] Tian F. Bradbury P. J. Brown P. J. Hung H. Sun Q., 2011 Genome-wide association study of leaf architecture in the maize nested association mapping population. Nat. Genet. 43: 159–162. 10.1038/ng.746 21217756

[bib67] Tyler, L., S. J. Lee, N. D. Young, G. A. DeIulio, E. Benavente *et al.*, 2016 Population structure in the model grass is highly correlated with flowering differences across broad geographic areas. Plant Genome 9 10.3835/plantgenome2015.08.0074. 10.3835/plantgenome2015.08.0074 27898828

[bib68] Vogel J. P. Tuna M. Budak H. Huo N. Gu Y. Q., 2009 Development of SSR markers and analysis of diversity in Turkish populations of *Brachypodium* distachyon. BMC Plant Biol. 9: 88 10.1186/1471-2229-9-88 19594938 PMC2719641

[bib69] Wang M. Jiang N. Jia T. Leach L. Cockram J., 2012 Genome-wide association mapping of agronomic and morphologic traits in highly structured populations of barley cultivars. Theor. Appl. Genet. 124: 233–246. 10.1007/s00122-011-1697-2 21915710

[bib70] Wheeler T. von Braun J., 2013 Climate change impacts on global food security. Science 341: 508–513. Available at: http://science.sciencemag.org/content/341/6145/508.abstract. 23908229 10.1126/science.1239402

[bib71] Wicker T. Mayer K. F. X. Gundlach H. Martis M. Steuernagel B., 2011 Frequent gene movement and pseudogene evolution is common to the large and complex genomes of wheat, barley, and their relatives. Plant Cell 23: 1706–1718. Available at: http://www.plantcell.org/lookup/doi/10.1105/tpc.111.086629. 10.1105/tpc.111.086629 21622801 PMC3123954

[bib72] Wilson D. Jones J., 1982 Effect of selection for dark respiration rate of mature leaves on crop yields of Lolium perenne cv. S23. Ann. Bot. 49: 313–320. Available at: http://aob.oxfordjournals.org/content/49/3/313.short. 10.1093/oxfordjournals.aob.a086255

[bib73] Wilson P. B. Streich J. C. Borevitz J. O., 2015a Genomic diversity and climate adaptation in *Brachypodium*, pp. 107–127 in Genetics and Genomics of Brachypodium, edited by Vogel J. Springer International Publishing, Cham, Switzerland 10.1007/7397_2015_18

[bib74] Wilson P. B. Rebetzke G. J. Condon A. G., 2015b Of growing importance : combining greater early vigour and transpiration efficiency for wheat in variable rainfed environments. Funct. Plant Biol. 42: 1107–1115. 10.1071/FP15228 32480749

[bib75] Wilson P. B. Rebetzke G. J. Condon A. G., 2015c Pyramiding greater early vigour and integrated transpiration efficiency in bread wheat; trade-offs and benefits. F. Crop. Res. 183: 102–110. 10.1016/j.fcr.2015.07.002

[bib76] Winzeler M. McCullough D. E. Hunt L. A., 1988 Genotypic differences in dark respiration of mature leaves in winter wheat (Triticum aestivum L.). Can. J. Plant Sci. 68: 669–675. 10.4141/cjps88-080

[bib77] Woods D. P. Ream T. S. Amasino R. M., 2014 Memory of the vernalized state in plants including the model grass *Brachypodium* distachyon. Front. Plant Sci. 5: 99 10.3389/fpls.2014.00099 24723926 PMC3971174

[bib78] Woods D. P. Bednarek R. Bouché F. Gordon S. P. Vogel J. P., 2017 Genetic architecture of flowering-time variation in *Brachypodium* distachyon. Plant Physiol. 173: 269–279. Available at: http://www.ncbi.nlm.nih.gov/pubmed/27742753%0Ahttp://www.plantphysiol.org/lookup/doi/10.1104/pp.16.01178. 10.1104/pp.16.01178 27742753 PMC5210718

[bib79] Xu S., 2003 Theoretical basis of the Beavis effect. Genetics 165: 2259–2268. 14704201 10.1093/genetics/165.4.2259 PMC1462909

[bib80] Yano K. Yamamoto E. Aya K. Takeuchi H. Lo P. C., 2016 Genome-wide association study using whole-genome sequencing rapidly identifies new genes influencing agronomic traits in rice. Nat. Genet. 48: 927–934. Available at: http://www.nature.com/doifinder/10.1038/ng.3596. 10.1038/ng.3596 27322545

[bib81] Zheng X. Levine D. Shen J. Gogarten S. M. Laurie C., 2012 A high-performance computing toolset for relatedness and principal component analysis of SNP data. Bioinformatics 28: 3326–3328. 10.1093/bioinformatics/bts606 23060615 PMC3519454

